# Improvement of Temperature Performance of Singlemode-Multimode-Singlemode Fiber Structure

**DOI:** 10.3390/s22218262

**Published:** 2022-10-28

**Authors:** Rongxiang Zhang, Weiyu Wang, Jianfei Zhang, Yuhong Han, Tao Liu

**Affiliations:** 1 College of Physics Science and Technology, Hebei University, Baoding 071002, China; 2 Department of Electronic and Communication Engineering, North China Electric Power University, Baoding 071003, China

**Keywords:** singlemode-multimode-singlemode fiber structure, temperature performance, stability, sensitivity

## Abstract

A theoretical model for studying the temperature properties of singlemode-multimode-singlemode (SMS) fiber structure fabricated by absorptive multimode fiber (MMF) cladding is established. Moreover, an SMS-based temperature sensor is fabricated and experimentally demonstrated. Experimental results show that the dip wavelength of the transmission spectrum changes linearly with temperature, which is in good agreement with the simulated results obtained by using the model. Further, a comprehensive study of temperature characteristics affected by the thermo-optic effect, thermal expansion effect, and thermal effect of absorption characteristics is performed for SMS fiber optic structures with different refractive indexes, thermo-optic coefficients, and absorption properties of MMF cladding, MMF core diameters, and thermal expansion coefficients of packaging shell. According to the obtained rules, investigations are carried out into the thermal response of an SMS fiber structure resulting from combined thermal effects for temperature performance optimization. Excellent temperature stability with a temperature sensitivity of 0 pm/°C or good temperature sensitivity of −441.58 pm/°C is achieved accordingly.

## 1. Introduction

The singlemode–multimode–singlemode (SMS) fiber structure consisting of two identical single-mode fibers (SMFs) axially spliced at both ends of a multimode fiber (MMF) has the advantages of simple structure, ease of fabrication, and low cost. It has been successfully utilized to sense refractive index (RI) [1,2,3], strain or pressure [4,5,6], heart rate [7], temperature [8,9,10,11], and so on. In the SMS fiber structure, the light is launched into an SMF, then propagates to an MMF and becomes many excited modes which eventually couple back to another SMF and the mode interference occurs. According to the principle of mode interference, the output of the SMS fiber structure depends on the optical property (RI and absorption) and physical dimension (diameter and length) of the MMF. That is to say, the mode interference can be modulated by changing these parameters [12]. Based on this theory, the SMS fiber structure can be used to be a sensor. In order to extend the application of an SMS fiber structure, the MMF cladding can be replaced by some materials whose optical property is influenced by other parameters, such as magnetic field [13,14]. For these sensors, the substitute works as the MMF cladding. Therefore, the variation of the parameters acting on the MMF cladding can be expressed by the change of optical property of the MMF cladding and measured by the change of the output of the SMS fiber structure.

Because of the thermo-optic effect (TOE), the thermal expansion effect (TEE), and the thermal effect of absorption characteristic, the optical property and physical dimension of the MMF in an SMS fiber structure are temperature sensitive, so the modes excited in the MMF vary with the change of temperature, and hence the output of the SMS fiber structure is correspondingly sensitive to temperature. Thus, the performance of the sensors based on the SMS fiber structure should be related to the temperature. The reported studies about the temperature characteristics of SMS fiber structure can be categorized into two types of purpose. One is to fabricate a temperature sensor with good sensitivity [8,9,10]. The other is to carry out the temperature compensation for a non-temperature sensor with the purpose of improving temperature stability [11]. Nonetheless, the temperature properties of the SMS fiber structure presented by the aforesaid studies are only for the cases with some fixed parameters. In our previous works [15], the temperature cross-sensitivity characteristics of SMS fiber structure packaged by a shell were studied. But the absorption characteristic of the MMF cladding was not considered, the effect of MMF core diameter on the temperature characteristics of the SMS fiber structure was not studied, and the temperature stability of the non-temperature sensor was not discussed either. Thus, no theoretical model for analyzing the temperature characteristics of the SMS fiber structure while taking into account the absorption characteristic of the MMF cladding has been proposed. As well, the temperature characteristics of the SMS fiber structure induced by all kinds of thermal effects are not analyzed detailed, and regular conclusions are not provided. In this paper, a theoretical model for studying the temperature properties of an SMS fiber structure fabricated by absorptive MMF cladding was established by analyzing the multimode interference principle and identifying temperature influence factors and their relations to temperature. Then an SMS fiber structure based temperature sensor was fabricated and experimentally demonstrated. The experimental results agreed well with theoretical results. Further, the effects of various factors, such as TOE, TEE, and thermal effect of absorption characteristics, on the temperature characteristics of the SMS fiber structure with different RIs, thermo-optic coefficients (TOCs), and absorption properties of MMF cladding, MMF core diameters, and thermal expansion coefficients (TECs) of packaging shell were investigated by using the proposed model. Finally, the optimal parameters of SMS fiber structure to improve the temperature stability and sensitivity were provided.

## 2. Theoretical Model

**Figure 1:**
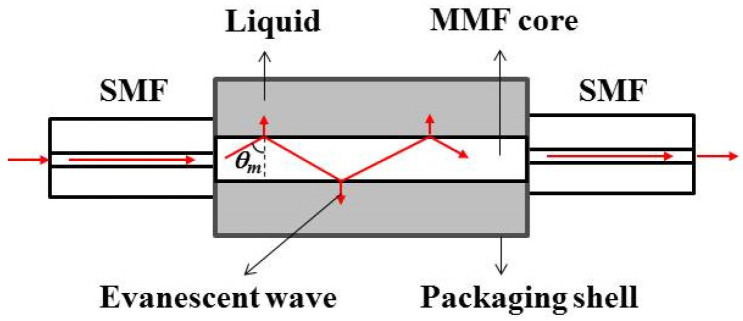
Schematic of SMS fiber structure.

Figure 1 shows the schematic diagram of an SMS fiber structure that is formed by splicing a segment of step-index MMF between two standard SMFs. The MMF section is totally sealed in a packaging shell that is filled with liquid. The light is launched into the input SMF with an approximate Gaussian-shaped field distribution. We assume that the axes of the SMFs and the MMF are perfectly aligned. In such a case, when light of fundamental mode *LP*_01_ within the SMF comes to the MMF, only a few circularly symmetric modes *LP*_0*m*_ are excited [1]. The light field in MMF can be regarded as the superposition of these excited modes, that is

(1) $$E\left(r,z\right)=\sum_{m=1}^{M}\Psi_{m}\left(r,z\right)$$

where *M* is the total number of excited modes, *r* is the radial coordinate of fiber, *z* is the propagation distance, and $\Psi_{m}\left(r,z\right)$ is the optical wave field function of each mode, which can be obtained by using the separation variable method [16]:

(2) $$\Psi_{m}\left(r,z\right)=\left\{\begin{array}{ll} c_{m}J_{0}\left(u_{m}\frac{r}{a_{co}}\right)e^{-i\beta_{m}z} & r\leq a_{co}\\ d_{m}K_{0}\left(w_{m}\frac{r}{a_{co}}\right)e^{-i\beta_{m}z} & r>a_{co} \end{array}\right.$$

(3) $$u_{m}=a_{co}\sqrt{k_{0}^{2}n_{co}^{2}-\beta_{m}^{2}}$$

(4) $$w_{m}=a_{co}\sqrt{\beta_{m}^{2}-k_{0}^{2}n_{cl}^{2}}$$

where *c_m_* and *d_m_* are excitation coefficients of higher-order modes, *J*_0_ represents the 0-order Bessel function, and *K*_0_ represents the 0-order Hankel function. The *a_co_* is the core radius of MMF, *k*_0_ is the wave number in a vacuum, *n_co_* and *n_cl_* are the RI of the MMF core and cladding, respectively, *β_m_*, *u_m_* and *w_m_* are the longitudinal propagating constant in MMF, transverse propagating constant in MMF core and cladding, respectively. The *u_m_* and *w_m_* satisfy the relationship of $w_{m}=\sqrt{V_{m}^{2}-u_{m}^{2}}$, where $V_{m}=\left(2\pi a_{co}/\lambda \right)\sqrt{n_{co}^{2}-n_{cl}^{2}}$ is the normalized frequency of the MMF.

The *u_m_* and *w_m_* can be obtained from the characteristic equation satisfied by the above eigen scalar modes, then *β_m_* can be calculated by using Equations (3) or (4). According to the scalar mode theory, the power coupling coefficient of each conduction mode excited in MMF is [16]

(5) $$\eta_{m}=c_{m}^{2}={\left(\frac{\int_{0}^{\infty }E(r,0)F_{m}(r)rdr}{\int_{0}^{\infty }F_{m}(r)F_{m}(r)rdr}\right)}^{2}\approx \frac{2{\left(\frac{\varpi }{a_{co}}\right)}^{2}\exp\left[-\frac{1}{2}{\left(\frac{\varpi }{a_{co}}\right)}^{2}u_{m}^{2}\right]}{J_{1}^{2}\left(u_{m}\right)+\frac{K_{1}^{2}\left(w_{m}\right)}{K_{0}^{2}\left(w_{m}\right)}J_{0}^{2}\left(u_{m}\right)}$$

where E(r,0) is the light field at the junction (*z* = 0) of SMF and MMF, that is, Gaussian light field in SMF, *F_m_*(*r*) is the field profile of the *LP*_0*m*_ mode. The *J_n_* represents the *n*-order Bessel function, and *K_n_* represents the *n*-order Hankel function.

Meanwhile, the MMF cladding, which mainly affects the evanescent wave resulting from the total reflection of conduction mode at the core-cladding interface, is an important factor to modulate the output of an SMS fiber structure. When the MMF cladding is an absorbent material, not only its RI, but also its absorption property will affect the evanescent wave. Taking into account the absorption of MMF cladding, when the light with amplitude of *E*_0_ propagates in fiber for distance *z*, its amplitude will attenuate to

(6) $$E\left(z\right)=E_{0}\exp\left(-\gamma z\right)$$

where *γ* is the evanescent attenuation coefficient. For the case of meridional rays, the evanescent attenuation coefficient of the m-order mode is given by [17]

(7) $$\gamma_{m}=\frac{\alpha \lambda n_{cl}\cos\theta_{m}\cot\theta_{m}}{4\pi a_{co}n_{co}{}^{2}\cos^{2}\theta_{c}\sqrt{\sin^{2}\theta_{m}-\sin^{2}\theta_{c}}}$$

where *λ* is the free space wavelength of light launched into the fiber, *α* is the absorption coefficient of MMF cladding at *λ*, *θ_c_* is the critical angle [*θ_c_* = sin^−1^(*n_cl_*/*n_co_*)], *θ_m_* is the angle of the ray with respect to the normal to the core-cladding interface in the sensing region.

Therefore, when the MMF cladding is an absorbent material, the field distribution at the length *z* in MMF can be written as [14]

(8) $$E(r,z)=\sum_{m=1}^{M}c_{m}F_{m}(r)\exp(i\beta_{m}z)\exp\left(-\gamma_{m}z\right)$$

Finally, these modes are coupled into the output SMF, as a result of interference, the transmittance of SMS structure can be expressed as [13]

(9) $$T(\lambda )=\sum_{m,n=1}^{M}c_{m}^{2}\cdot c_{n}^{2}\cdot \cos\left[(\beta_{m}-\beta_{n})L\right]\cdot \exp[-(\gamma_{m}+\gamma_{n})L]$$

where *L* is the length of MMF. The transmittance *T* expressed by Equation (9) is a function of wavelength, so if broadband light containing many wavelengths is input into the SMS fiber structure, the transmission spectrum will be obtained from the output of the SMS fiber structure. According to Equations (1)–(9), any variation of the following parameters, the RIs of the MMF core and cladding (*n_co_* and *n_cl_*), the radius and length of the MMF core (*a_co_* and *L*), and the absorption coefficient of the MMF cladding (*α*) will cause a change in the longitudinal propagating constant *β*, or the excitation coefficient *c*, or the evanescent attenuation coefficient *γ* of each excited mode in MMF, resulting in the changes of *T* and dip wavelength of the transmission spectrum. Meanwhile, when any of the above parameters are different, the change amplitude and trend of dip wavelength with temperature will also be different.

For an SMS fiber structure as shown in Figure 1, the changes of RIs of the MMF core and cladding induced by the TOE, the changes in the diameter and length of the MMF core induced by the TEE, the changes of the RI, diameter, and length of the MMF core induced by the axial strain caused by different TECs of packaging material (*τ_p_*) and MMF core (*τ_co_*) can be respectively described by

(10) $$\Delta n_{co\_1}=\xi_{co}\times \Delta T$$

(11) $$\Delta n_{cl}=\xi_{cl}\times \Delta T$$

(12) $$\Delta d_{1}=d_{0}\times \tau_{co}\times \Delta T$$

(13) $$\Delta l_{1}=l_{0}\times \tau_{co}\times \Delta T$$

(14) $$\Delta n_{co\_2}=-\frac{n_{co\_0}^{3}}{2}\left[p_{12}-\nu \left(p_{11}+p_{12}\right)\right]\times \left(\tau_{p}-\tau_{co}\right)\times \Delta T$$

(15) $$\Delta d_{2}=-\nu \times d\times \left(\tau_{p}-\tau_{co}\right)\times \Delta T$$

(16) $$\Delta l_{2}=l\times \left(\tau_{p}-\tau_{co}\right)\times \Delta T$$

where ∆*T* is the change of temperature, *ξ_co_* and *ξ_cl_* are the TOC of the MMF core and cladding, respectively, *d*_0_ and *l*_0_ are the diameter and length of the MMF core at 25 °C, respectively, *n_co_*__0_ is the RI of the MMF core at 25 °C, *p*_11_ and *p*_12_ are elastic-optic coefficients, *ν* is the Poisson ratio. For silica fibers, *p*_11_ = 0.12, *p*_12_ = 0.27, and *ν* = 0.17 [18]. 

Furthermore, the absorption coefficient of the MMF cladding is related to the change in temperature, which is defined as:

(17) $$\Delta \alpha =\delta_{\alpha }\times \Delta T$$

where *δ_α_* is the change rate of absorption coefficient with the temperature.

From the above, the RIs of the MMF core and cladding, diameter and length of the MMF core, and absorption coefficient of the MMF cladding can be expressed as

(18) $$n_{co}=n_{co\_0}+\Delta n_{co\_1}+\Delta n_{co\_2}$$

(19) $$n_{cl}=n_{cl\_0}+\Delta n_{cl}$$

(20) $$d=d_{0}+\Delta d_{1}+\Delta d_{2}$$

(21) $$l=l_{0}+\Delta l_{1}+\Delta l_{2}$$

(22) $$\alpha =\alpha_{0}+\Delta \alpha$$

where *n_cl_*__0_ and *α*_0_ are the RI and absorption coefficient of the MMF cladding at 25 °C, respectively. Based on Equation (9), in which the parameters are determined by Equations (1)–(8) and (10)–(22), the transmission spectrum of the SMS fiber structure at a certain temperature can be achieved. Subsequently, the temperature response of the SMS fiber structure, that is, the change of dip wavelength with temperature can be obtained from the transmission spectra at different temperatures. As a result, the theoretical model given in Section 2 can be used to investigate the individual or combined effect of all kinds of thermal effects on the temperature characteristics of the SMS fiber structure with different parameters. 

## 3. Experimental and Simulated Results

**Figure 2:**
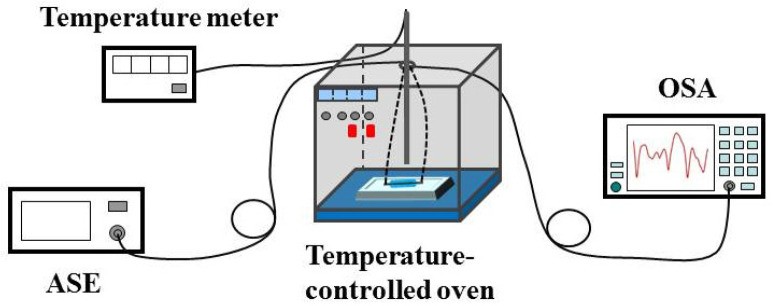
Experimental setup.

**Figure 3:**
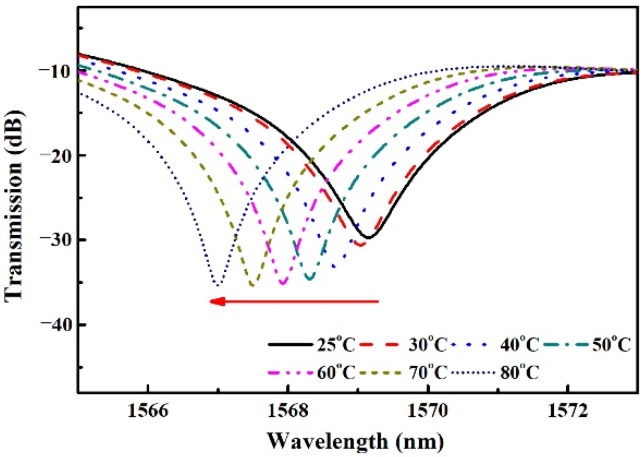
Transmission spectra at different temperatures.

In this work, an SMS fiber temperature sensor is designed. The production and measurement process of the temperature response of the sensor shown in Figure 1 are the same as our previous work [15]. The SMF and MMF are the standard SMF-28 (Corning Inc., New York, NY, USA) and commercialized no-core fiber (NCF, Prime Optical Fiber Co., Taiwan, China), respectively. The core diameter and numerical aperture of SMF are 8.2 μm and 0.14, respectively. The NCF is made of pure silica. The diameter, length, TOC, and TEC of the NCF are 61.5 μm, 8.2 cm, 1.06 × 10^−5^/°C, and 5 × 10^−7^/°C, respectively [18]. The glass capillary with an approximate TEC of 8 × 10^−6^/°C [19], an inner diameter of 0.5 mm, and a length of 10 cm is filled with distilled water. The distilled water serves as the cladding of the NCF. The RI, TOC, and absorption coefficient of distilled water are 1.32, −1.5 × 10^−4^/°C, and 10^3^/m, respectively [20,21,22]. The change rate of the absorption coefficient of distilled water with the temperature is about −5/m/°C [23]. The experimental setup and the measured transmission spectra are shown in Figure 2 and Figure 3, respectively. From Figure 3 we can see that an interference dip with good visibility appears at 1569.16 nm at 25 °C and shifts towards a shorter wavelength with the increase in temperature.

**Figure 4:**
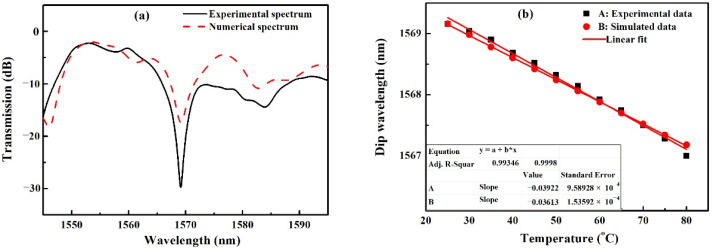
Compare of the numerical and experimental results: (**a**) Transmission spectra at 25 °C; (**b**) Variation of dip wavelength of the spectrum with the temperature.

Based on the theoretical background in Section 2, the numerical simulation is carried out by using the mode propagation analysis (MPA) method. The MMF core is made of pure silica and its RI is calculated by the Sellmeier equation [18]. The other parameters of the sensor are chosen according to the parameters of the materials employed in our experiment as described above. The simulation results present a similar change for the transmission spectra of the sensor, that is, the interference dip shifts to the shorter wavelength with the increase in temperature. Figure 4 shows the comparison of the numerical and experimental results. Figure 4a depicts the transmission spectra at 25 °C and Figure 4b shows the relationships between the dip wavelength and temperature. We can see that the simulated result is in good agreement with the experimental result. The dip wavelength changes linearly as the temperature increases and the linear fitting results present nearly the same sensitivity of −39.22 pm/°C and −36.13 pm/°C, respectively, for the experiment and simulation. Thus the correctness of the theoretical model and simulation is verified.

The temperature characteristics of an SMS sensor obtained from the experiment are the combined results induced by various thermal effects of TOE, TEE, and absorption characteristics. It is difficult to identify the individual role of each thermal effect and effectively control the temperature property by choosing or adjusting parameters in the SMS fiber structure. Thus in the following works, we utilize the numerical simulation to investigate the impact of various thermal effects on the temperature characteristics of an SMS fiber structure separately and comprehensively, with the purpose of improving temperature stability and sensitivity.

## 4. Impact of MMF on the Temperature Performance of SMS Fiber Structure

### 4.1. Effect of TOE of MMF Cladding (TOE_cl_)

**Figure 5:**
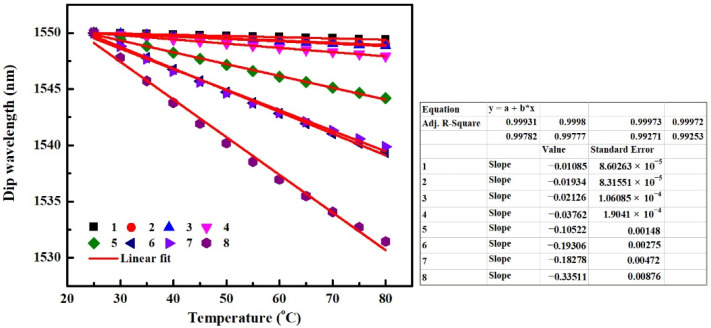
Dip wavelength as a function of the temperature for SMS fiber structures, only the TOE_cl_ is taken into account in the simulation.

In order to study only the effect of TOE_cl_ on the temperature characteristics of an SMS fiber structure, the other thermal responses of the MMF and packing are not taken into account here. Some related parameters of MMF are shown in Table 1, the other parameters of MMF and the parameters of SMF which are not illustrated in Table 1, and the parameters which are not listed in other tables in the following sections either, are all the same as those in Section 3. Since the sensitivity demodulated from the wavelength shift of an SMS structure is independent of the MMF length and proportional to the dip wavelength [2], an identical dip wavelength of 1550 nm at 25 °C is obtained for various SMS structures in the following works. When only the TOE_cl_ is taken into account in simulation, the obtained results of dip wavelength that decreases with the increase of the temperature for SMS fiber structures with different parameters corresponding to Table 1 are depicted in Figure 5. 

From Figure 5, it can be seen that the dip wavelengths have linear relationships with the temperature for all SMS fiber structures, but the temperature sensitivities are different. This result indicates that the effect of TOE_cl_ on the temperature characteristics of an SMS fiber structure is related to the RI of MMF cladding (RI_cl_), TOC of MMF cladding (TOC_cl_), and MMF core diameter. As can be seen from Figure 5, when the RI_cl_ increases from 1.32 to 1.42, the temperature sensitivity increases to nearly 9~10 times of its initial value by comparing the cases of “1, 2, 3, 4” with “5, 6, 7, 8”, respectively. When the TOC_cl_ changes from −1 × 10^−4^/°C to −2 × 10^−4^/°C, the temperature sensitivity increases to about 2 times its initial value by comparing the cases of “1, 2, 5, 6” with “3, 4, 7, 8”, respectively. Furthermore, when the MMF core diameter decreases from 105 μm to 60 μm, the temperature sensitivity increases to nearly two times its initial value by comparing the cases of “1, 3, 5, 7” with “2, 4, 6, 8”, respectively. The above results demonstrate that negative temperature sensitivity can be enhanced by increasing the value of RI_cl_ or TOC_cl_, or decreasing the MMF core diameter, and vice versa. Moreover, the RI_cl_ plays a major role in determining the effect of TOE_cl_.

**Figure 6:**
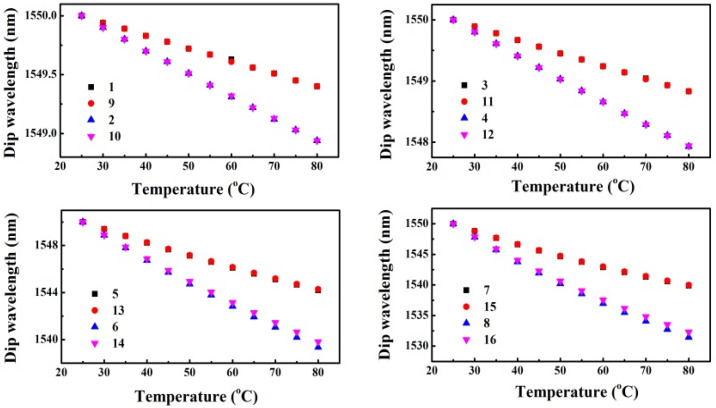
Dip wavelength as a function of the temperature for SMS fiber structures, only the TOE_cl_ is taken into account in the simulation.

To study the influence of the absorption coefficient of MMF cladding over the effect of TOE_cl_ further, similar simulations are carried out by only changing the absorption coefficient of MMF cladding from 10^3^/m to 10^4^/m for the SMS fiber structures represented by the numbers “1~8” in Table 1. To avoid confusion, the SMS fiber structures with α of 10^4^/m are represented by numbers “9~16” corresponding to “1~8”, respectively. Figure 6 shows the dip wavelengths as a function of temperature for the SMS fiber structures “1~16”. We can see that the absorption coefficient of MMF cladding almost has no influence on the effect of TOE_cl_ by comparing the cases of “1~8” with “9~16”, respectively. That is, the temperature sensitivity is almost constant for SMS fiber structures with identical parameters except for the absorption coefficient of MMF cladding.

### 4.2. Effect of TOE of MMF Core (TOE_co_)

**Figure 7:**
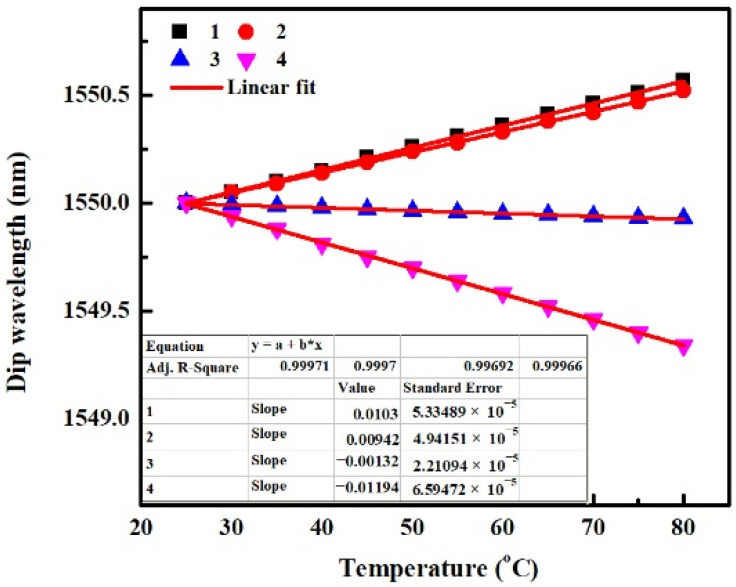
Dip Wavelength as a function of the temperature for SMS fiber structures, only the TOE_co_ is taken into account in the simulation.

Similar to the analysis in Section 4.1, here we study only the effect of TOE_co_ on the temperature characteristics of an SMS fiber structure. The related parameters of MMF are shown in Table 2. The dip wavelength is simulated as a function of temperature in Figure 7.

It can be seen from Figure 7 that the dip wavelengths are dependent on temperature linearly for all SMS fiber structures, but the direction and magnitude of sensitivities are diverse, which means the effect of TOE_co_ is related to the RI_cl_ and MMF core diameter that affect the transmittance *T* of SMS fiber structure based on Equation (9) and relevant Equations (1)–(8). When the RI_cl_ varies from 1.32 to 1.42, the temperature sensitivity changes from positive value to negative value by comparing the cases of “1, 2” with “3, 4”, respectively. Moreover, when the MMF core diameter changes from 105 μm to 60 μm, the positive temperature sensitivity decreases for smaller RI_cl_ of 1.32, whereas the negative temperature sensitivity increases for bigger RI_cl_ of 1.42, by comparing the cases of “1, 3” with “2, 4”, respectively. Thus positive temperature sensitivity can be amplified by decreasing the RI_cl_ or increasing the MMF core diameter, while negative temperature sensitivity can be enhanced by increasing the RI_cl_ or decreasing the MMF core diameter. Additionally, it can also be found that the absorption coefficient of MMF cladding almost has no influence on the effect of TOE_co_ by comparing the dip wavelengths at different temperatures for SMS fiber structures with identical parameters except for the absorption coefficient of MMF cladding.

### 4.3. Effect of TEE

**Figure 8:**
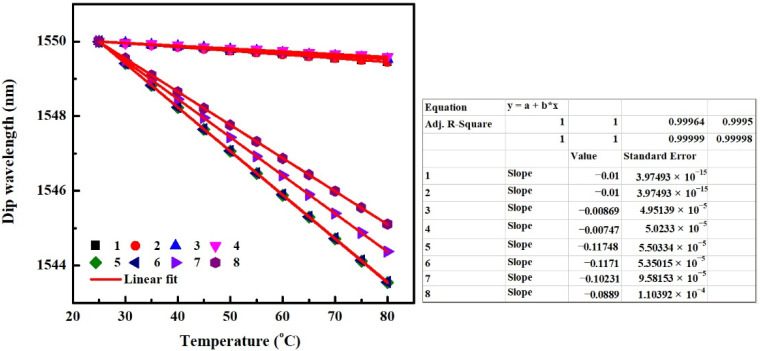
Dip Wavelength as a function of the temperature for SMS fiber structures, only the TEE is taken into account in the simulation.

For the purpose of studying the effects of TEEs of the MMF core (TEE_co_) and packaging material (TEE_p_) on the temperature characteristics of an SMS fiber structure, it is supposed that the other thermal responses of the MMF don’t take effect. The related parameters of MMF and packaging shell are shown in Table 3. When only the TEE_co_ and TEE_p_ are taken into account, the simulated results of dip wavelength at different temperatures are shown in Figure 8. 

It can be seen from Figure 8 that the dip wavelengths change still linearly with temperature for all SMS fiber structures, but the sensitivities are not identical. This result indicates that the effects of TEE_co_ and TEE_p_ depend on the TEC of packaging material (TEC_p_), RI_cl,_ and MMF core diameter. When the TEC_p_ changes from 5 × 10^−6^/°C to 5 × 10^−5^/°C, the negative temperature sensitivity increases by comparing the SMS fiber structures “1, 2, 3, 4” with “5, 6, 7, 8”, respectively. When the RI_cl_ varies from 1.32 to 1.42, the negative temperature sensitivity is almost unchanged for smaller TEC_p_ of 5 × 10^−6^/°C, whereas that decreases for the bigger TEC_p_ of 5 × 10^−5^/°C, by comparing the SMS fiber structures “1, 2, 5, 6” with “3, 4, 7, 8”, respectively. Furthermore, when the MMF core diameter changes from 105 μm to 60 μm, the negative temperature sensitivity remains almost unchanged for the smaller TEC_p_ of 5 × 10^−6^/°C and the bigger TEC_p_ of 5 × 10^−5^/°C with smaller RI_cl_ of 1.32, as shown as the cases of “1–6” in Figure 8. While for bigger TEC_p_ of 5 × 10^−5^/°C with bigger RI_cl_ of 1.42, the negative temperature sensitivity decreases with the decrease of MMF core diameter, as shown in the cases of “7” and “8” in Figure 8. The above results indicate that the negative temperature sensitivity can be improved by increasing the TEC_p_ or MMF core diameter, or decreasing RI_cl_. Moreover, the TEC_p_ plays a major role in determining the effects of TEE_co_ and TEE_p_. Similarly, one can also find that the absorption coefficient of MMF cladding almost has no influence on the effects of TEE_co_ and TEE_p_ by comparing the dip wavelengths at different temperatures for SMS fiber structures with identical parameters except for the absorption coefficient of MMF cladding.

### 4.4. Effect of Thermal Effect of Absorption Characteristic of the MMF

**Figure 9:**
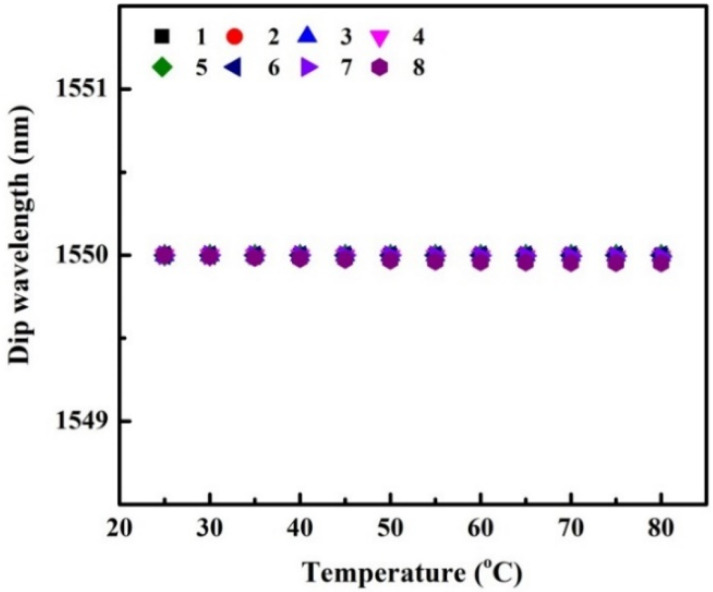
Dip Wavelength as a function of the temperature for SMS fiber structures, only the thermal effect of absorption characteristic is taken into account in the simulation.

In this section, we only investigate the impact of the absorption effect of the MMF cladding on the temperature characteristics of an SMS fiber structure. The related parameters of MMF are shown in Table 4. The obtained results of dip wavelength that changes with the increase of temperature are shown in Figure 9. 

As can be seen from Figure 9, the dip wavelengths of all SMS structures at different temperatures are almost constant. So we can conclude that the absorption effect of the MMF cladding has barely an influence on the thermal sensitivity of an SMS structure. 

## 5. Improvement of Temperature Performance

### 5.1. Improvement of Temperature Stability

**Figure 10:**
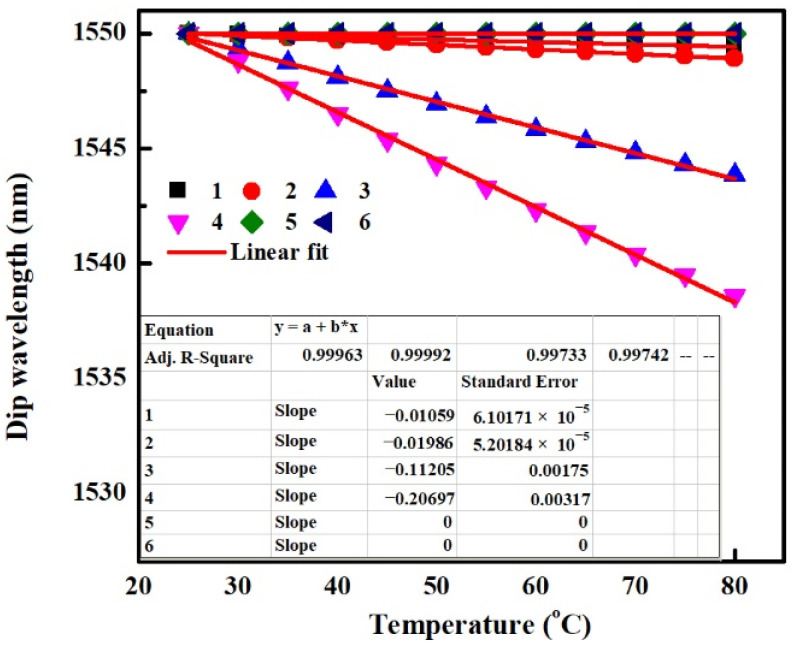
Dip Wavelength as a function of the temperature for SMS fiber structures, various thermal effects are all taken into account in the simulation.

For the non-temperature sensor based on SMS fiber structure, it is crucial to eliminate the temperature disturbance. According to the above results, it can be concluded that the negative temperature sensitivity can be decreased by decreasing the value of TOC_cl_ or TEC_p_. However, as RI_cl_ increases or MMF core diameter decreases, temperature sensitivity induced by TOE and TEE changes oppositely. Hence the thermal responses of SMS fiber structures with identical TOC_cl_ of −1 × 10^−4^/°C and TEC_p_ of 5 × 10^−6^/°C but different RI_cl_s and MMF core diameters are analyzed. The parameters of MMF and packaging shell are shown in Table 5 for different SMS fiber structures. The obtained temperature sensitivities taking into account the combined effect of the TOEs and TEEs are shown in Figure 10. 

It can be seen that the temperature sensitivity of −10.59 pm/°C for SMS fiber structure “1” is the smallest one among SMS fiber structures “1–4”. Considering that small TEC_p_ is conducive to decreasing the negative temperature sensitivity, the dip wavelength is simulated as a function of temperature for SMS fiber structure with small TEC_p_ of 5 × 10^−7^/°C in Figure 10. This case is marked “5” shown in Table 5. The linear fitting result shows that the temperature sensitivity for the case of “5” is 0 pm/°C, which means that the SMS fiber structure in this case has very good thermal stability. Thus, SMS fiber structure “5” shown in Table 5 is recommended to be used as a non-temperature sensor. In order to analyze the influence of the absorption effect of the MMF cladding, the dip wavelength that changes with the increase of temperature for SMS fiber structure without considering the absorption of MMF cladding (shown in the case of “6” in Table 5) is also obtained and shown in Figure 10. The result again shows that the thermal sensitivity of an SMS structure is almost independent of the absorption effect of the MMF cladding.

### 5.2. Improvement of Temperature Sensitivity

**Figure 11:**
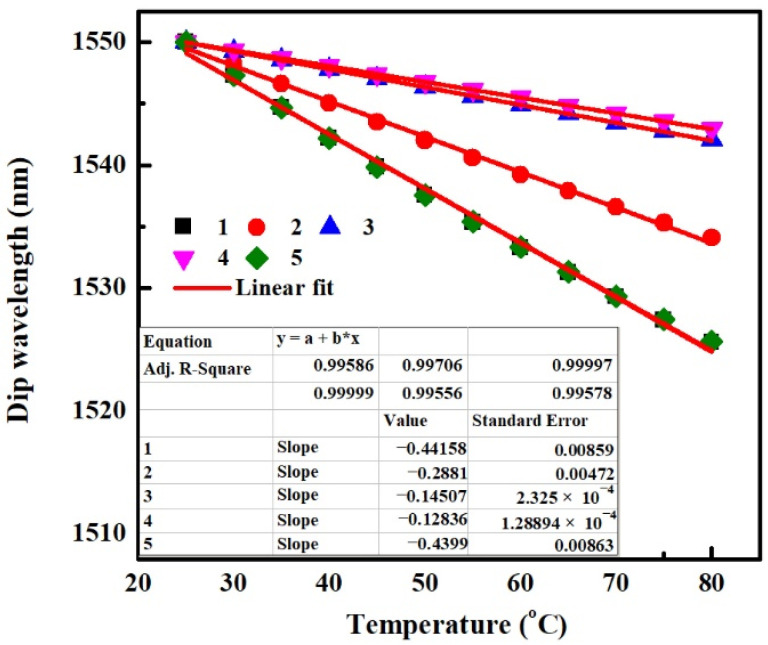
Dip Wavelength as a function of the temperature for SMS fiber structures, various thermal effects are all taken into account in the simulation.

For the SMS fiber structure-based temperature sensor, the higher the temperature sensitivity the better the performance of the sensor. According to results obtained from Section 4, it can be seen that the negative temperature sensitivity can be enhanced by increasing the value of TOC_cl_ or TEC_p_. But similarly, as RI_cl_ increases or MMF core diameter decreases, the change of temperature sensitivity induced by TOE and TEE is the opposite. So to achieve high temperature sensitivity, the thermal responses of SMS fiber structure with the same TOC_cl_ of −2 × 10^−4^/°C and TEC_p_ of 5 × 10^−5^/°C but different RI_cl_s and MMF core diameters are investigated. The parameters of MMF and packaging shell are shown in Table 6. The obtained temperature sensitivities taking into account the combined effect of the TOEs and TEEs are shown in Figure 11. As can be seen, the temperature sensitivity of −441.58 pm/°C for the SMS fiber structure “1” is the biggest one among SMS fiber structures “1–4”. Thus, the SMS fiber structure “1” is recommended to be used as a temperature sensor. From Figure 11 we can also see that, the absorption effect of the MMF cladding still has little influence on the thermal sensitivity of an SMS structure, by comparing the SMS structure “1” with “5” whose parameters are the same as those of “1” except that the *α* and *δ_α_* are both set to 0.

In our previous work [15], glycerol–water mixture (3:1 volume mixture) with a RI of 1.43 and a TOC of −1.827 × 10^−4^/°C served as MMF cladding. While in this paper, distilled water with a RI of 1.32 and a TOC of −1.5 × 10^−4^/°C serves as the MMF cladding. Except that, the other experimental materials are the same in both sensors of previous and current works. The temperature sensitivity of −453.4 pm/°C and −39.22 pm/°C are obtained, respectively. These results indicate that the greater the values of RI_cl_ and negative TOC_cl_, the greater the value of negative temperature sensitivity, and vice versa, which is in agreement with the rules of the theoretical research shown in Figure 10 and Figure 11.

## 6. Conclusions

Because of the TOE, TEE, and thermal effect of absorption characteristics, the performance of an SMS based sensor is related to the temperature. In this paper, the impacts of various thermal effects on the temperature properties of an SMS fiber structure are studied in detail. Firstly, a theoretical model is established by analyzing the multimode interference principle and identifying temperature influence factors and their relations to temperature. This model is suitable for the SMS fiber structure fabricated by absorptive MMF cladding. Then an SMS based sensor is designed and experimentally demonstrated. The experimental results show the transmission spectrum of the sensor is affected by temperature and the dip wavelength has a linear relationship with the temperature, which agrees well with the simulated results based on the proposed model. Subsequently, the temperature characteristics of an SMS fiber optic structure affected by the TOE, TEE, and thermal effect of absorption characteristics are analyzed comprehensively by using the theoretical model. The results show that for the SMS fiber structure, (1) the negative temperature sensitivity induced by TOE_cl_ can be amplified by increasing the value of RI_cl_ or TOC_cl_, or decreasing the MMF core diameter; (2) the positive temperature sensitivity induced by TOE_co_ can be improved by decreasing the RI_cl_ or increasing the MMF core diameter, while the negative temperature sensitivity induced by TOE_co_ can be enhanced by increasing the RI_cl_ or decreasing the MMF core diameter; (3) the negative temperature sensitivity induced by TEE_co_ and TEE_p_ can be enhanced by increasing the TEC_p_ or MMF core diameter, or decreasing RI_cl_; (4) the absorption effect of the MMF cladding does not have a significant influence on the thermal sensitivity of the SMS structure. Finally, based on the above results, a study of temperature characteristics affected by combined thermal effects is carried out for temperature performance optimization of an SMS fiber optic structure. Excellent temperature stability with a temperature sensitivity of 0 pm/°C or good temperature sensitivity of −441.58 pm/°C can be obtained by optimizing the parameters of the SMS fiber structure.

## Figures and Tables

**Table 1:** Parameters of MMF in the SMS fiber structure.

Number of SMS Structure	*α* (/m)	RI_cl_	TOC_cl_ (/°C)	*d* (μm)
1	10^3^	1.32	−1 × 10^−4^	105
2	10^3^	1.32	−1 × 10^−4^	60
3	10^3^	1.32	−2 × 10^−4^	105
4	10^3^	1.32	−2 × 10^−4^	60
5	10^3^	1.42	−1 × 10^−4^	105
6	10^3^	1.42	−1 × 10^−4^	60
7	10^3^	1.42	−2 × 10^−4^	105
8	10^3^	1.42	−2 × 10^−4^	60

**Table 2:** Parameters of MMF in the SMS fiber structure.

Number of SMS Structure	*α* (/m)	RI_cl_	*d* (μm)
1	10^3^	1.32	105
2	10^3^	1.32	60
3	10^3^	1.42	105
4	10^3^	1.42	60

**Table 3:** Parameters of MMF and packaging shell in the SMS fiber structure.

Number of SMS Structure	*α* (/m)	TEC_p_ (/°C)	RI_cl_	*d* (μm)
1	10^3^	5 × 10^−6^	1.32	105
2	10^3^	5 × 10^−6^	1.32	60
3	10^3^	5 × 10^−6^	1.42	105
4	10^3^	5 × 10^−6^	1.42	60
5	10^3^	5 × 10^−5^	1.32	105
6	10^3^	5 × 10^−5^	1.32	60
7	10^3^	5 × 10^−5^	1.42	105
8	10^3^	5 × 10^−5^	1.42	60

**Table 4:** Parameters of MMF in the SMS fiber structure.

Number of SMS Structure	*α* (/m)	*δ_α_* (/m/°C)	RI_cl_	*d* (μm)
1	10^3^	−5	1.32	105
2	10^3^	−5	1.32	60
3	10^3^	−5	1.42	105
4	10^3^	−5	1.42	60
5	10^3^	−50	1.32	105
6	10^3^	−50	1.32	60
7	10^3^	−50	1.42	105
8	10^3^	−50	1.42	60

**Table 5:** Parameters of MMF and packaging shell in the SMS fiber structure.

Number of SMS Structure	*α* (/m)	*δ_α_* (/m/°C)	TOC_cl_ (/°C)	TEC_p_ (/°C)	RI_cl_	*d* (μm)
1	10^3^	−5	−1 × 10^−4^	5 × 10^−6^	1.32	105
2	10^3^	−5	−1 × 10^−4^	5 × 10^−6^	1.32	60
3	10^3^	−5	−1 × 10^−4^	5 × 10^−6^	1.42	105
4	10^3^	−5	−1 × 10^−4^	5 × 10^−6^	1.42	60
5	10^3^	−5	−1 × 10^−4^	5 × 10^−7^	1.32	105
6	0	0	−1 × 10^−4^	5 × 10^−7^	1.32	105

**Table 6:** Parameters of MMF and packaging shell in the SMS fiber structure.

Number of SMS Structure	*α* (/m)	*δ_α_* (/m/°C)	TOC_cl_ (/°C)	TEC_p_ (/°C)	RI_cl_	*d* (μm)
1	10^3^	−50	−2 × 10^−4^	5 × 10^−5^	1.42	60
2	10^3^	−50	−2 × 10^−4^	5 × 10^−5^	1.42	105
3	10^3^	−50	−2 × 10^−4^	5 × 10^−5^	1.32	60
4	10^3^	−50	−2 × 10^−4^	5 × 10^−5^	1.32	105
5	0	0	−2 × 10^−4^	5 × 10^−5^	1.42	60
